# Delusion of Pregnancy in Down Syndrome: Two Case Reports

**DOI:** 10.3390/ijerph192013339

**Published:** 2022-10-16

**Authors:** Luciana Ursumando, Elisa Fucà, Floriana Costanzo, Stefano Vicari

**Affiliations:** 1 Child and Adolescent Neuropsychiatry Unit, Department of Neuroscience, Bambino Gesù Children’s Hospital, IRCCS, 00146 Rome, Italy; 2 Department of Life Sciences and Public Health, Catholic University, 00168 Rome, Italy

**Keywords:** intellectual disability, psychiatric disorders, psychosis, trisomy 21

## Abstract

Individuals with intellectual disability (ID) are more vulnerable to psychotic disorder and schizophrenia than the general population. However, psychotic symptoms have not been widely described in this population. Here, we deeply investigated the cases of two young women with ID and Down syndrome (DS) who developed a delusion of pregnancy, a rare condition defined as a fixed belief of being pregnant despite factual evidence to the contrary. The assessment included psychopathological and neuropsychological examination, as well as the evaluation of cognitive and adaptive functioning. In these cases, delusion manifested as a psychotic symptom of a cyclothymic disorder (case 1) or as an independent delusional disorder (case 2). However, some similarities emerged: both women exhibited good pre-morbid adaptive functioning and family history of psychiatric disorders; moreover, in both cases delusion emerged in association with an external trigger. Difficulties in verbally expressing one’s thoughts and beliefs were found, as well as poor abstract reasoning skills that may have affected the ability to deeply conceptualize the delusional idea itself. These findings may provide crucial insights into the clinical manifestation of psychosis in individuals with DS and underscore the importance of a routine psychological and neuropsychological follow-up to provide prompt and adequate intervention.

## 1. Introduction

Delusion of pregnancy is defined as a fixed belief of being pregnant despite factual evidence to the contrary: absence of physical signs and symptoms suggestive of pregnancy and negative confirmatory tests [1]. According to the Diagnostic and Statistical Manual of Mental Disorders-5 (DSM-5), delusion of pregnancy is an unspecified type of delusional disorder that falls within the spectrum of schizophrenia and other psychotic disorders, and may or may not have bizarre content: “delusions are deemed bizarre if they are clearly implausible, not understandable, and not derived from ordinary life experience” [2]. In addition, it may be transient or long-lasting. Cases with delusion of pregnancy lasting 15 years [3], 18 years [4] and 20 years [5] have been described.

Delusion of pregnancy has often been reported in women of reproductive age who suffer or have suffered from a psychiatric illness in the past [6], but there are some isolated cases in males as well [7,8,9,10,11]. Although it is considered a rare condition, it is quite common in developing countries (e.g., India or sub-Saharan Africa) where there is strong cultural pressure on women to be fertile [12].

The etiology is heterogeneous and involves both biological and psychological factors. The range of biological explanations includes organic disorders such as epilepsy, hyponatremia, hypothyroidism, metabolic syndrome, and hyperprolactinemia induced by antipsychotics or other organic cause [13]. Psychological factors include cognitive misinterpretation of bodily sensations and physical changes, poor reality testing and wish-fulfilment, psychological distress and cultural pressures on women to have children, which may be present concomitantly [14].

The literature on delusion of pregnancy includes mainly case reports. A systematic review by Bera and Sarkar [14] included 84 cases in which the most common diagnoses were schizophrenia (35.7%), bipolar disorders (16.7%) and depression (9.5%). Cipriani and Fiorino [15] investigated the symptoms of delusion of pregnancy in the context of dementia, hypothesizing that this phenomenon may be an early presentation of frontotemporal dementia, which is often misdiagnosed as schizophrenia.

Although delusion of pregnancy falls within the spectrum of schizophrenia and other psychotic disorders, it has not been widely described in people with intellectual disability (ID), which are more vulnerable to psychopathological disorders [16,17].

Epidemiological studies suggest that about 40% of people with ID exhibit psychopathological disorders [18,19], with a total prevalence higher than that of the general population [16], even if the reported prevalence rate varies widely among studies [19]. Of note, the prevalence of psychiatric symptoms is higher across neurogenetic syndromes (e.g., Down syndrome and Prader–Willi syndrome), ranging from 32% to 74% [20]. A recent meta-analysis [17] reported an increased risk of unspecified psychotic disorder and schizophrenia in people with ID, compared with a risk in the general population. Conversely, the prevalence rates of other psychopathological disorders (mood disorders, anxiety disorder and personality disorders) were found to be lower when compared to general population [17].

Although people with ID would seem to be more vulnerable to psychiatric disorders, it is still difficult to recognize and treat them [21,22]. The capability of self-report symptoms may be limited by communicative and linguistic difficulties [23,24,25] as well as by other visual, attentive and neuropsychological deficits [21,26]. For instance, discriminating hallucinations from developmentally appropriate behaviors such as talking to imaginary friends could be problematic in individuals with ID [27]. On the other hand, aggression or increase in self-injury could be atypical clinical presentations of a mood disorder in this population [26]. In addition, there is a paucity not only of standardized instruments for the psychopathological assessment [28], but also of randomized controlled trials to evaluate the effects of psychopharmacological treatment in people with ID [22,29]. As a result, adequate diagnosis and treatment could be hampered [26].

To the best of our knowledge, the only report describing the presence of delusions of pregnancy in people with ID is dated back to 1989 [7]. The author described a case of delusions of pregnancy in a man with moderate ID, epilepsy and psychosis; the man had hallucinatory symptoms (he heard many voices calling to him) and reported the feeling that someone was pressing his chest and that he had a baby in his abdomen. However, the author stated that ID and epilepsy seem to be the most likely causative factor in the development of delusion of pregnancy, rather than co-occurring psychotic disorder.

Considering the lack of described cases of delusion of pregnancy in people with ID, here we deeply investigated two cases of young women with ID and Down syndrome (DS) who developed a delusion of pregnancy.

DS is the most frequent genetic condition associated with ID, caused by the presence of an additional copy of chromosome 21 [30]. DS is characterized by low intelligence quotient (IQ), in the range of moderate to severe ID, associated with deficits in language, memory and executive functions [31,32,33], and significant psychopathology (18–23%) [34].

Although the prevalence of psychopathology and behavioral problems in people with DS is higher than in the general population [32], this prevalence is lower than individuals with ID of other genetic etiology [34]. With regard to psychiatric symptoms such as hallucinations and delusions in DS, the level of baseline intellectual impairment does not inextricably influence psychiatric disorders [35]; moreover, Urv and colleagues [36] identified delusions or hallucinations in up to 79% of older adults with possible or overt dementia. However, mixed results were reported in a more recent study, which found no significant differences in the presence of delusions symptoms between people with DS with or without dementia [37]. It should be noted that the paucity of cases reported in literature may be related to the underestimation of delusions in DS. Indeed, only a case of erotomanic delusions in a 42-year-old woman with DS without dementia [38] and a case of mirrored-self misidentification delusion in a 53-year-old man with DS and Alzheimer’s Disease [39] have been reported.

Here, we described the cases of two young women who manifested delusion of pregnancy within a hypomania period (case 1) or as an independent delusional disorder (case 2). The young women underwent psychopathological, neuropsychological, cognitive and adaptive functioning assessment.

### 1.1. Clinical Case 1

When the young woman came to our observation, she was 29 years old. A family history of psychiatric disorders was reported, namely depression and a suicide attempt in a third-degree paternal relative and depression in the direct paternal line. The socioeconomic status of the family was middle class. The young woman was born at 39 weeks’ gestation by natural childbirth. Free trisomy 21 was diagnosed at birth; no pre-, peri-, or postnatal problems were reported; she had global developmental delay. Therefore, she underwent speech, physical and psychomotor therapy until the age of 10 years. She did not suffer from epilepsy or other neurological disorders.

Her language skills developed functionally, with relatively good morphosyntactic and lexical skills. The young woman attended school with the help of school support. Parents reported that she adapted relatively well to the school environment, parents described her as lively and hyper-social from an early age, although marked irritability emerged in adolescence. After finishing secondary school, she began working in a beauty salon and then in a hotel, under the supervision of an educator. She also participated on educational activities abroad, without significant difficulties in social interaction and autonomy.

Parents reported the onset of behavioral and emotional problems and the appearance of pregnant ideas in the previous six months. The young woman firmly believed and told her parents that she was expecting a child, refusing to accept the contradictory evidence they provided. The belief that she was pregnant apparently emerged after the young woman watched a video describing the pregnancy and cesarean delivery.

From that moment, she allegedly began having recurrent thoughts of wanting a baby and sensory misperceptions in her body, such as belly growth and abdominal movements. In addition, the young woman reported being afraid of being touched by imaginary people or that her family members might attack her belly. The parent also reported dysfunctional behaviors, such as a desire to undress, refusal to lie on the bed, sleep deprivation, and psychomotor agitation.

Four months after the onset of psychomotor agitation, tactile misperceptions, and the emergence of the delusional ideations, she was taken to an emergency psychiatric ward. She started drug therapy with zuclopenthixol 14 mg/day and biperiden 4 mg/day, with gradual improvement of symptoms.

The young woman came to our observation two months after the start of drug treatment. On arrival she was alert and responsive, although ideomotor slowing was present and she was poorly cooperative. On clinical psychiatric examination she presented with depressed mood and irritability. She no longer reported a belief that she was pregnant, although she was convinced that she had been pregnant in the past. No other bizarre behaviors or beliefs were present. No neurological symptoms were present.

Physical examination revealed the presence of elevated prolactin values (49.3 ng/mL) and hypothyroidism (FT3: 1.62 pg/mL, FT4: 0.73 ng/dL and TSH: 44.7 µIU/mL).

### 1.2. Clinical Case 2

When the young woman came to our observation, she was 17 years old. A family history of psychiatric disorders was reported, namely depression and schizophrenia in a fourth-degree maternal relative. The family’s socioeconomic status was middle class. She was the child of a pregnancy complicated by threatened miscarriage; she was born at 34 weeks’ gestation by caesarean delivery. At birth, she was diagnosed with free trisomy 21. She had global developmental delay; language skills emerged late, but communication was functional: she was able to produce short but complete sentences. She received speech, physical and psychomotor therapy until the age of 13. She did not suffer from epilepsy or other neurological disorders.

At age 10, she began attending psychotherapy for anxiety problems associated with daytime encopresis during social moments perceived as stressful (wedding parties, shopping at the supermarket); at the time of the visit, the encopresis episodes ceased and psychotherapy was discontinued.

At the time of the visit, the young woman is attending high school with school support. Her parents reported that she never had separation or socialization difficulties at school and had good autonomies at home: she helped them with small household chores (e.g., washing the dishwasher and making the bed).

The young woman came to our observation because over the past year she has externalized the belief that she was pregnant. According to parent reports, the onset of the belief occurred after learning of her aunt’s pregnancy. Since that time, the young woman began to spend a lot of time actively searching for pregnancy information, downloading pregnancy monitoring apps, pregnant mother and baby care games, and pictures of fetuses. In addition, the young woman reported sensory misperceptions related to the status of pregnancy, such as the perception of nausea, feeling the baby’s heartbeat in her belly, and the sensation of “water breaking”.

In addition, teachers reported that the young woman began to show marked interest and jealousy toward a classmate; she also created social profiles claiming to be her classmate.

At the time of our visit, the young woman was not taking any medication.

During the visit she was alert, responsive and cooperative and her mood was euthymic. Her social functioning was not markedly impaired and, with the exception of her belief that she was pregnant, she was not exhibiting bizarre or strange behaviors. No neurological symptoms were present.

Physical examination ruled out hyperprolactinemia (prolactin: 15.4 ng/mL) and hypothyroidism (FT4: 0.91 ng/dL and TSH: 0.91 µIU/mL).

## 2. Materials and Methods

### 2.1. Cognitive and Adaptive Functioning

The cognitive profile was assessed using Leiter International Performance Scale-Third Edition (Leiter-3) [40]. The Leiter-3 is a completely nonverbal assessment in which neither examiner nor examinee has to speak; in fact, it is widely used with people with speech difficulties. The test can be administered from age 3 years old to over 75 years of age.

The Leiter-3 focuses on fluid intelligence, and the Cognitive Scale is composed of the following subtests: Figure Ground (FG), Classification and Analogies (CA), Sequential Order (SO), Form Completion (FC), and Matching/Repeated patterns (optional subtest). The administration takes about 45 min.

We used the Italian version and, for each subtest, raw scores were converted into normalized scaled scores, using Italian reference norms. The sum of the four subtest-scaled scores, preferably FG, FC, CA, and SO, provided a global nonverbal IQ (nvIQ).

Adaptive functioning was measured through the Adaptive Behavior Assessment System (ABAS II) [41], a standardized caregiver questionnaire developed to measure a wide range of daily adaptive skills for individuals ages 0–89 years.

Adaptive behavior is measured at several levels, including a General Adaptive Composite (GAC) divided into three derived domains (Conceptual, Social and Practical) that incorporate nine individual adaptive skill areas. For GAC and adaptive domains, composite scores are converted to standard scores, while for individual adaptive skill areas, raw scores are converted to scaled scores.

### 2.2. Psychopathological Examination

The young women underwent a neuropsychiatric examination, including clinical interviews and direct observations, by a team of neuropsychiatrists and clinical psychologists to investigate the presence of past (lifetime) and current psychopathological symptoms. Clinical interviews were used to investigate the possible presence of psychiatric disorders according to DSM-5 criteria. The interviews were conducted by well-trained clinicians with strong clinical experience and extensive knowledge about psychopathology and neurodevelopmental disorders. Both parents and young women were considered as a source of information. If general symptoms emerged, detailed questions were used to verify the diagnosis.

To assess the level of impairment in a person’s general functioning, the Global Assessment of Functioning (GAF) scale [42] was used. This scale reflects the level of symptom severity considering psychological, social and occupational functioning on a hypothetical continuum between mental health and illness. Scores range are from 100 to 1, divided into ten ranges, where scores from 100 to 60 indicate adequate functioning, scores from 60 to 40 the presence of obvious problems, scores from 40 to 30 the presence of severe problems, and scores below 30 a severe/extreme impairment.

### 2.3. Neuropsychological Evaluation

#### 2.3.1. Verbal Fluency

The verbal fluency test, taken from the NEPSY II neuropsychological battery [43], assesses the ability to quickly generate words according to semantic and phonemic categories. It consists of two subtests: semantic fluency and phonological fluency. The semantic fluency subtest includes two one-minute tests in which participants are asked to pronounce animal names and food and drink names, respectively. The phonological fluency task requires participants to pronounce as many words as possible that begin with the letter “S” in the first case and the letter “F” in the other. The raw scores were converted into age-equivalent scores by comparing them with available normative values in order to provide an estimate of the chronological age at which a typically developing child demonstrates the indicated skills (e.g., scaled score 10).

#### 2.3.2. Verbal Short-Term Memory

The sentence repetition test, taken from the NEPSY II neuropsychological battery [43], assesses verbal short-term memory: the participants is read a series of sentences and asked to recall each sentence immediately after its presentation. The raw scores were converted into age-equivalent scores by comparing them with available normative tables, to provide an estimate of the chronological age at which a typically developing child demonstrates the indicated skills (e.g., scaled score 10).

#### 2.3.3. Visual-Motor Integration

To assess visual-motor integration, the Beery-Buktenica Developmental Test of Visual-Motor Integration (Beery VMI) was used [44]. The Beery VMI consists of geometric drawings of increasing difficulty; participants are asked to observe the geometric drawings and copy them with pencil and paper. Scores are based on how accurately the drawings were copied: higher scores indicate greater visual-motor ability. Raw scores were converted into age-equivalent scores according to available standards.

#### 2.3.4. Academic Skills

Reading was assessed through the decoding test, taken from the Assessment of Reading and Comprehension Skills for Elementary and Middle School (MT-3 Clinical Tests) [45]. The person is asked to read aloud a text with a time limit of 4 min; reading time (in seconds per syllable) and accuracy (number of errors) are assessed.

To assess mathematical skills, three tests from the Test for the Assessment of Computation and Problem-Solving Skills (AC-MT 6–11) were used [46]: mental calculation, which examines the ability to apply computational procedures (the child must solve addition and subtraction); number judgment, to assess semantic understanding of numerical quantities (for several pairs of Arabic numerals, the child must identify the largest number); number ordering, to assess the semantic representation of numbers (the child must write a random series of numbers displayed in descending or ascending order). The raw scores were compared with normative tables from first-grade, corresponding to an age-equivalent academic level of 6–7 years.

## 3. Results

### 3.1. Case 1

#### 3.1.1. Cognitive and Adaptive Functioning

Moderate ID emerged. The Leiter-3 nonverbal cognitive scale was administered, and the young woman obtained a score below normal range, corresponding to a nvIQ of 45 (<1st percentile). The cognitive profile appeared to be in line with the adaptive functioning profile as measured by the ABAS-II (GAC score: 57, <1st percentile; Conceptual score: 59, <1st percentile; Social score: 64, <1st percentile; and Practical score: 40, <1st percentile).

#### 3.1.2. Psychopathological Examination

Based on the psychopathological interview and DSM-5 criteria [2], a diagnosis of cyclothymic disorder (301.13; F34.0) with psychotic symptoms was made. The psychopathological interview revealed the presence of hypomania and depressive periods in the past two years. Depressive symptoms were present in the previous two months, namely: irritability, apathy and anhedonia, motor slowdown, marked decrease in interests, fatigue and lack of energy. In addition, declines in memory and attentional functions, easy distractibility and poverty of speech were reported.

Six months prior to our visit, the young woman began to manifest symptoms of hypomania characterized by expansive and irritable mood, hypertrophic self-esteem, distractibility, accelerated thinking, feeling of being watched, nocturnal awakenings and insomnia. These symptoms rapidly increased, and a four-month-long monothematic delusion of pregnancy emerged, characterized by recurrent thoughts of wanting a child and bodily misperceptions related to pregnancy status. She was firmly convinced that she was pregnant despite reasonably contradictory evidence as to its veracity. Visual and tactile misperceptions related to the theme of delusion were present.

The bizarre characteristics of the delusional themes could not be defined, since the young woman was unable to provide explanation of conception.

Overall, the young woman was given a GAF score of 31, indicating the presence of significant functional impairment and inability to function in certain situations, such as at home, at school, with peers, and in the general social context.

#### 3.1.3. Neuropsychological Evaluation

At the verbal fluency test the young woman obtained a raw score of 11 in semantic fluency, corresponding to an age-equivalent score of 3 years, and a raw score of 5 in phonological fluency, corresponding to an age-equivalent score much less than 7 years. At the verbal short-term memory test, she obtained a raw score of 10, corresponding to an age-equivalent score of 4 years and 11 months. Finally, at the visual-motor integration test she obtained a raw score of 15, corresponding to an age-equivalent score of 6 years and 6 months.

It should be noted that during administration the young woman proved tired, uncooperative and reluctant to continue and, as for the academic tests, the decision was made to discontinue the test. Her poor cooperation may have been at least in part affected her performance on neuropsychological testing.

### 3.2. Case 2

#### 3.2.1. Cognitive and Adaptive Functioning

Mild ID was found to be present. On the Leiter-3 nonverbal cognitive scale the young woman obtained a score below normal range, corresponding to a nvIQ of 56 (<1st percentile) but in line with the adaptive functioning profile, as measured by the ABAS-II (GAC score: 56, <1st percentile; Conceptual score: 59, <1st percentile; Social score: 58, <1st percentile; and Practical score: 61, <1st percentile).

#### 3.2.2. Psychopathological Examination

Based on the psychopathological interview and DSM-5 criteria [2], the diagnosis of delusional disorder (297.1; F22) was made. The psychopathological interview revealed the presence of a monothematic delusion of pregnancy lasting one year. No hallucinations or symptoms attributable to schizophrenia were present; although erroneous tactile perceptions such as perceived nausea, heartbeat sensation, and “water breaking” were reported, these were related to the delusional theme. The young woman was firmly convinced that she was pregnant, despite reasonably contradictory evidence as to its veracity.

The onset of a subthreshold depressive episode lasting about 1 month, characterized by loss of interest in most activities, irritable mood, increase appetite, and insomnia, preceded the onset of delusion. However, the independent diagnosis of delusional disorder was confirmed because the pregnancy belief emerged after the remission of depressive symptoms; moreover, the depressive episode was shorter than the duration of the delusional period.

With the exception of the pregnancy belief, the young woman’s functioning did not appear markedly impaired and her behavior did not appear bizarre or strange. She maintained intact psychosocial functioning, with the exception of her marked interest in a classmate, which manifested itself in odd behaviors, such as jealousy and creating social profiles in which she claimed to be her. However, this did not seem to be part of the delusion of pregnancy. These behaviors lasted a few weeks and they were modulated and reduced by the environmental context, parents and teachers.

Overall, the young woman was given a GAF score of 50, indicating the presence of an overt problem in only one area.

#### 3.2.3. Neuropsychological Evaluation

At verbal fluency test, the young woman obtained a raw score of 27 in semantic fluency, corresponding to an age-equivalent score of 8 years, and a raw score of 11 in phonological fluency, corresponding to an age-equivalent score of 7 years. At the verbal short-term memory test, she obtained a raw score of 8, corresponding to an age-equivalent score of 4 years and 6 months. At the visual-motor integration test, the young woman obtained a raw score of 15, corresponding to an age-equivalent score of 6 years and 6 months. On academic tests, reading skills were shown in the normal range for a first-grade child (age range 6–7 years), both in reading time (2.26 s per syllable; 90th percentile) and accuracy (1 error; 80th percentile). Similarly, mathematical skills were found to be in the normal range for a first-grade child (age range 6–7 years), considering mental calculation (3 correct answers out of 6; −0.75 z score), number judgment (6 correct answers out of 7; +0.43 z score) and number ordering (8 correct answers out of 10; +0.54 z score) tasks.

## 4. Discussion

The purpose of this study was to describe for the first time two cases of delusion of pregnancy in individuals with DS. To the best of our knowledge, no cases of delusion of pregnancy in people with DS have been described in the literature. More generally, the literature on this form of delusion in people with ID is scarce, despite the fact that this population is more vulnerable to psychotic disorders [17].

We characterized the two cases in terms of psychopathology, neuropsychological, and cognitive and adaptive functioning. This assessment allowed us to identify similarities and differences between the two cases.

Assessment of the cognitive profile revealed similar abilities between the two girls: both have mild to moderate ID, with good pre-morbid adaptive functioning, similar to what has been previously described in acute neuropsychiatric disorders in DS [47]. We identified adequate adaptive abilities, especially in the social and language areas. However, the neuropsychological performance slightly differed between the two young women on some verbal abilities, because case 1 showed poorer verbal fluency and did not complete academic skills evaluation. Conversely, they similarly performed below cognitive level on verbal short-term memory and showed similar visual-motor integration skills, on average for their cognitive level, around 6 years and 6 months of age. This neuropsychological profile is in line with literature on DS [32,48,49]. Case 2 also showed academic skills in line with 6–7 years of mental age. They both had no comorbidities with other neurodevelopmental and neurological disorders. Of note, both young women had a family history of psychopathological disorders. This indicates that genetic vulnerability may play a significant role in the etiopathogenesis of psychotic disorders even in neurogenetic syndromes, such as DS.

In terms of the clinical manifestation of the delusion, the two cases also presented similarities: the delusion of pregnancy arose after a direct exposure to information about pregnancy, as an external trigger. In fact, in case 1 the young woman had seen a video of a cesarean delivery, and in case 2 the young woman had learned about her aunt’s pregnancy, and received explanations about her pregnancy status. However, the parents nor young women did not report any psychological antecedents to the delusion, such as social isolation, desire to be treated as a pregnant woman or to compensate for childlessness, and difficulty accepting being infertile, which are often described in people without ID [6]. Only in case 2, the psychological antecedent may be traced in a form of “copy-cat” effect, whereby the young woman may have initially manifested a kind of emulation of her aunt’s pregnancy, and, subsequently, she may have begun to develop symptoms of delusion [50,51,52]. Thus, in this case, both external and internal triggers (the desire to be like her aunt) seem to be present. However, this was not explicitly stated by the young woman, but it was reported exclusively by her parents.

The initial circumstances of the onset and development of delusion of pregnancy in the two cases resemble the stages of delusion described by Conrad in his works on psychosis manifestation [53]. Previous reports have shown that the nature of the psychotic disorders in individuals with ID did not significantly differ from those without ID [54]. More specifically, both young women initially expressed a desire to have a child and began actively seeking pregnancy information (step 1). Subsequently, they have reported the onset of some sensory neutral perceptions (stage 2), such as abdominal movement or nausea. These sensations were gradually replaced by pregnancy-specific sensations, such as a growing belly (case 1), or the sensation of feeling the baby’s heartbeat in the belly or “water breaking” (case 2), which are usually read as a confirmation of pregnancy (step 3). However, it is not possible to definitively clarify whether the two women misinterpreted sensory perceptions as signs of pregnancy or if they just referred these symptoms in absence of specific sensory inputs, because their ability to verbalize on symptoms was limited. Moreover, unlike most of the cases reported in the literature, including reports in psychosis [14] or dementia [15], the two young women were not able to provide any explanation (plausible or otherwise) concerning their pregnancy cause and how conception had occurred.

Difficulties in deeply characterizing symptoms are frequently encountered in the assessment of people with ID, as it is done through verbal self-report [55,56]. However, the clinical manifestation of delusion of pregnancy in our two cases clearly indicates that the initiation and development of the delusional idea is analogue to that shown in population without ID, while the delusional idea appeared to be poor in contents and details.

Verbal expression is known to be poorer in individuals with ID, however, even if verbal, individual with ID’s thought content may also be different from those without ID [26]. Therefore, another possible explanation for poor contents and details of the delusional idea could be ascribed to low abstract reasoning skills in our two cases, which may affect the ability to deeply conceptualize the delusional idea itself.

Regarding the etiology of the delusion of pregnancy, we found differences between the two cases: in case 1 the delusion of pregnancy appeared within a hypomanic symptomatology, although the attribution of grandiose components to the delusional idea was not present; in case 2, on the other hand, the delusion of pregnancy appeared as an independent delusional disorder.

Furthermore, from a physiological point of view, a possible association between hypothyroidism and hyperprolactinemia and the onset of delusion of pregnancy could be postulated in case 1. Cases of delusion of pregnancy with elevated prolactin have been reported in the literature [12,57,58]. However, in case 1, the detected hyperprolactinemia was likely to be antipsychotic-induced, whereas the delusion onset occurred six months before the visit, when the young woman was drug-free, and the delusion ended after two months of drug treatment. Since information on the prolactin values at the time of delusion onset was not available, a clear relation between hyperprolactinemia and delusion of pregnancy cannot be established in case 1. Nevertheless, the etiology of delusion of pregnancy is heterogeneous, and therefore hyperprolactinemia cannot clearly explain this delusion in all individuals; indeed, in case 2, prolactin levels were, on the contrary, in the normal range.

Overall, the two cases showed several similarities and a clear difference in the associated conditions. Case 1 represents an example of delusion of pregnancy in an individual with a diagnosis of cyclothymic disorder with psychotic symptoms, in line with most diagnoses described in the literature [14]. On the other side, case 2 is an independent disorder not associated with other psychiatric disorders, which appears to be a rarer condition.

Similarities with the population without ID are shown for the onset and development of the delusional idea, while differences seem to emerge on the conceptualization of the delusional idea.

Our cases highlight the need for careful consider psychiatric disorders in people with DS and other forms of ID. The fact that both young women had a family history of psychiatric disorder underscores the crucial role of adequate family history assessment in evaluating individuals with ID, possibly by multiple informants. Researchers agree that a range of psychopathological disorders and emotional problems are possible in comorbidity with ID and DS [59]. Therefore, mental health services and professionals need such evidence to better understand how these disorders occur and how to assess them in population with ID, in order to develop appropriate knowledge and skills about the needs of people with ID [21].

Verbal dysfunction and cognitive and attentional deficits in individuals with ID may result in a reduced capacity to deal with complex social problems and may predispose to psychotic disorders [54]. Individuals with ID exhibit more difficulty coping with stressful events; for instance, it has been reported that they utilize more avoidant coping strategies to deal with stressful interactions [60,61]. This could be due, at least in part, to the fact that cognitive efforts to face negative emotions require complex metacognitive skills, such as abstract thinking and focused attention, resulting in difficulty for children with ID [62]. An appropriate psychopathological and neuropsychological evaluation is crucial to provide adequate services that take into account special needs of individuals with ID. This encompasses not only healthcare, but also forensics and legal services which must strike a sometimes delicate balance between protecting these people from harm and improving their independence in life in order to participate in society as fully as possible [63]. Therefore, the individual perspective should be taken into account in the evaluation of psychopathological issues in ID in order to develop of adequate mental-health and social systems [64,65].

## 5. Conclusions

The present in-depth description of delusion of pregnancy in two young women with DS is of great importance in providing appropriate care for individuals with DS. To the best of our knowledge, this is the first reported cases of delusion of pregnancy in young women with DS associated with cyclothymic disorder and as an independent form of delusional disorder, respectively. Psychopathological, neuropsychiatric, neuropsychological, and cognitive and adaptive functioning information was provided to better characterize the profile of the two cases. The evaluation found both similarities and differences between the described cases. In fact, both women had good pre-morbid adaptive functioning and both had family history of psychiatric disorders. In addition, external triggers to the onset of delusional ideation could be identified in both cases. Globally, difficulties in verbally expressing one’s thoughts and beliefs and poor abstract reasoning skills emerged, which may have affected the ability to deeply conceptualize the delusional ideations itself. However, it should be noted that delusion of pregnancy can occur in various psychopathological conditions even in individuals with DS. Overall, these reports suggest the importance of a multifaceted approach that takes into account both behavioral and cognitive symptoms for people in whom psychiatric symptoms occur in association with genetic disorders, such as DS, and low cognitive and neuropsychological abilities. Further studies are also needed to better understand the genetic and environmental factors that explain psychotic disorders in DS.

## Data Availability

The data presented in this study are available on request from the corresponding author. The data are not publicly available due to privacy and ethical restrictions.
